# Epx4 Nanopore With Multiple Constrictions for Single‐Molecule Identification

**DOI:** 10.1002/smtd.70762

**Published:** 2026-06-11

**Authors:** Ayako Ijuin, Kota Naito, Mana Sato, Virginia Di Toro Mammarella, Nanami Takeuchi, Mauro Chinappi, Yoshikazu Tanaka, Ryuji Kawano

**Affiliations:** ^1^ Department of Biotechnology and Life Science Tokyo University of Agriculture and Technology (TUAT) Koganei‐shi Tokyo Japan; ^2^ Graduate School of Life Sciences Tohoku University Sendai Miyagi Japan; ^3^ Department of Industrial Engineering University of Rome Tor Vergata Roma Italy

**Keywords:** nanopore, nanotechnology, sensing applications

## Abstract

Nanopore technology enables rapid, portable, and label‐free single‐molecule detection of analytes, including nucleic acids and proteins. One strategy for improving accuracy is to increase interactions between analytes and the nanopore. For example, engineered nanopores with additional constrictions improve analyte‐pore interactions during translocation. This concept adopts the exploration of biological nanopores, which naturally contain multiple constrictions. Here, we demonstrate that Epx4, a pore‐forming toxin with two independent β‐barrels, functions as a nanopore sensor capable of generating informative ionic current signals during polypeptide translocation. Structural analysis of the pore geometry revealed that Epx4 contains up to four constrictions. In single‐molecule measurements, Epx4 detected cationic polypeptides with a higher event frequency than α‐hemolysin (αHL). With machine‐learning‐assisted analysis, Epx4 achieved an ROC AUC score of 0.82 and an F1 score of 0.72, both of which were higher than those obtained with αHL. Our findings suggest that Epx4 is a promising candidate for developing nanopore sensors with high accuracy for protein analysis.

## Graphical abstract

Epx4 is a biological multi‐constriction nanopore that generates informative current signals for polypeptide sensing. Compared with α‐hemolysin, Epx4 detects short poly‐L‐lysine (S‐PLL, Mw 10 kDa) at a higher event frequency and produces signal features that distinguish it from long poly‐L‐lysine (L‐PLL, Mw 30–70 kDa), with an ROC AUC of 0.82 for the single‐molecule identification.

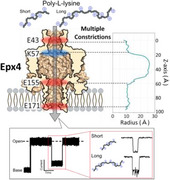

## 1 Introduction

Nanopore technology has emerged as a powerful tool for rapid, portable, and label‐free single‐molecule detection. When a nanopore is reconstituted in a bilayer lipid membrane (BLM), ions flow through the pore. As target molecules pass through the nanopore, the ionic current is temporarily blocked, generating characteristic blocking signals. These signals reflect molecular properties such as size, structure, and surface charge, enabling identification or sequencing based on characteristic current patterns. Since the first demonstration of single‐stranded DNA (ssDNA) and RNA detection using α‐hemolysin (αHL) in 1996 [1], this technology has advanced rapidly to enable DNA sequencing. The commercial release of nanopore sequencers by Oxford Nanopore Technologies in 2014 was a significant milestone in this advancement [2]. Nanopores remain the most promising candidates for driving the next developments in single‐molecule proteomics [3]. Various biological nanopores have been explored as nanopores capable of identifying target proteins, such as αHL [4, 5], curlin sigma S‐dependent growth subunit G pore (CsgG) [3], cytotoxin K (CytK) [6], fragaceatoxin C (FraC) [7, 8, 9, 10], aerolysin (AeL) [5, 11, 12, 13, 14], and *Mycobacterium smegmatis* porin A (MspA) [15, 16, 17, 18].

One strategy to improve sensing resolution is to increase interactions between the pore and the analyte. For example, the engineered CsgG and CsgF complex introduces a second constriction within the pore, improving the reading accuracy of homopolymer ssDNA by increasing interaction sites [19, 20]. The presence of two constrictions (N17 and Y51/N55/F56) creates multiple interaction points along the pore, leading to more complex and informative current signals. Such complex signals can be analyzed using machine‐learning‐based basecalling tools [19]. In addition to such an engineered nanopore, AeL has been reported to contain multiple constrictions and to generate characteristic blocking signals arising from interactions with molecules translocating through the pore [21, 22]. Extending this concept motivates the search for new biological nanopores that intrinsically have multiple constriction sites without requiring additional engineering [23].

**FIGURE 1:**
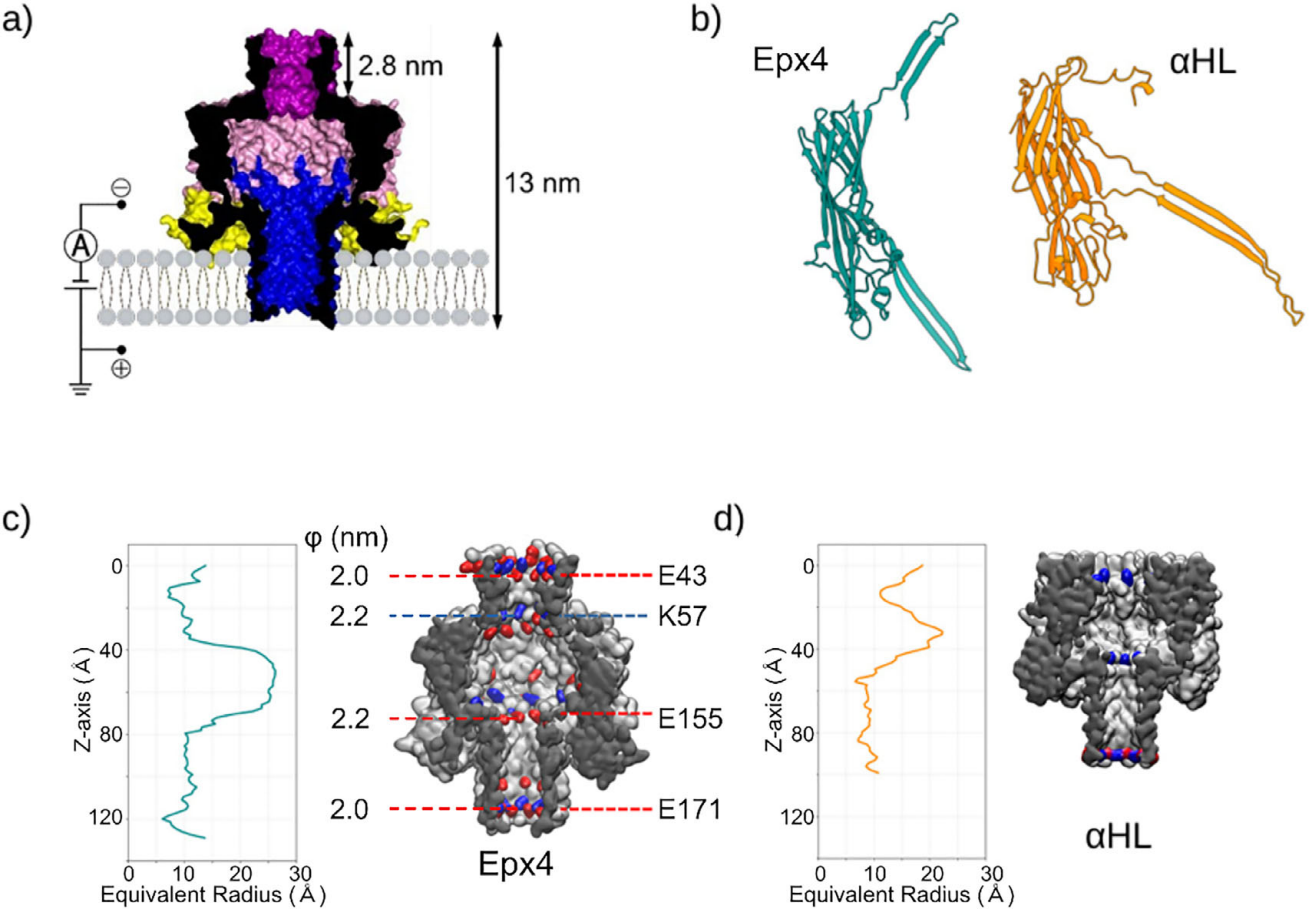
Structural features and pore dimensions of Epx4 and αHL. (a) Cross‐section structure of the Epx4 nanopore. The protein assembly is organized into four distinct domains: top (magenta), cap (pink), rim (yellow), and stem (blue). (b) Structural comparison of the individual protomers of Epx4 (light blue) and αHL (orange). (c) Pore radius profile of Epx4 (PDB: 7T4D) calculated via POVME3.0 analysis using a 1.5 Å grid spacing. The equivalent radius is plotted as a function of the *z*‐axis. The molecular model highlights the residues defining the main constrictions: E43, K57, E155, and E171. The diameters of each constriction were calculated from POVME3.0 results. (d) POVME3.0 analysis of the αHL pore (PDB: 7AHL) using a 1.5 Å grid spacing, showing the radial distribution along the channel. The narrowing regions correspond to residues K8, K147, K131, and D128. In the surface representations, acidic and basic residues at the constrictions are highlighted in red and blue, respectively.

*Enterococcus* pore‐forming toxin 4 (Epx4) is a homo‐octameric transmembrane pore‐forming toxin (PFT) composed of four structural domains: the top, cap, rim, and stem (Figure 1a). Epx4 assembles into an octameric pore containing two β‐barrel structures: a stem β‐barrel in the transmembrane region and an additional β‐barrel in the extramembrane top domain [24]. Previous studies have shown that Epx4 induces lysis of lipid membranes composed of POPC, PE, and cholesterol, and that charged residues in the top domain contribute to SDS‐resistant oligomer formation and cytotoxicity in HeLa cells without substantially altering overall structure [24] (Text S1). These findings establish Epx4 as a structurally distinct β‐barrel pore‐forming toxin.

Many β‐barrel PFTs used in nanopore sensing, such as AeL, αHL, and CytK, possess sensing regions within their β‐barrel structures [25], where constriction sites play a central role in signal generation. Because Epx4 contains two β‐barrels, each with potential constriction sites, it may provide multiple interaction sites for translocating molecules and generate more complex current signals. This study tests the hypothesis that a naturally occurring multi‐constriction nanopore can enhance signal information content and improve molecular discrimination, even under conditions with limited chemical diversity. In this study, we evaluated the potential of Epx4 as a nanopore sensor for single‐molecule detection. First, we identified internal constrictions within Epx4 using molecular dynamics (MD) simulations and characterized its electrophysiological properties through channel current measurements. Next, we assessed the sensing capability of Epx4 using homopolymer ssDNA and homopolymeric cationic polypeptides. We then evaluated its ability to discriminate polypeptides using multiple machine‐learning‐based classification methods.

## 2 Methods

### 2.1 Reagents and Chemicals

The reagents used in this study were as follows: KOD SYBR qPCR Mix (TOYOBO Co., Ltd., Osaka, Japan); NucleoSpin Gel and PCR Clean‐up (Takara Bio Inc., Shiga, Japan); SYBR Green I (Takara Bio Inc.); XL‐DNA Ladder KE‐2510 (Apro Science Group / Pharma Foods International Co., Ltd., Kyoto, Japan); Purefrex 2.0 (GeneFrontier, Chiba, Japan); Dynabeads His‐Tag Isolation and Pulldown (Thermo Fisher Scientific K.K., Tokyo, Japan); magnesium acetate tetrahydrate (Mg(OAc)_2_·4H_2_O; Kishida Chemical Co., Ltd., Osaka, Japan); tris(hydroxymethyl)aminomethane (Tris; Nacalai Tesque, Kyoto, Japan); hydrochloric acid (HCl; FUJIFILM Wako Pure Chemical Industries, Ltd. (Wako), Osaka, Japan); imidazole (Wako); sodium lauryl sulfate (SDS; Nacalai Tesque); EzRunT (ATTO, Tokyo, Japan); EzStandard LMW (ATTO); Prolite Orange (AAT Bioquest, Inc., Pleasanton, CA, USA); 2‐mercaptoethanol (Wako); glycerol (Wako); p‐PAGEL mini‐slab gel (ATTO); EzRun (ATTO); EzApply (ATTO); polymethyl methacrylate plate (PMMA; Mitsubishi Rayon Co., Ltd., Tokyo, Japan); 2‐dioleoyl‐sn‐glycero‐3‐phosphocholine (DOPC; Avanti Polar Lipids, Inc., Alabaster, AL, USA); parylene C (Polychloro‐p‐xylylene; Parylene Japan, Tokyo, Japan); 1,2‐diphytanoyl‐sn‐glycero‐3‐phosphocholine (DPhPC; Avanti Polar Lipids, Inc.); *n*‐decane (Wako); potassium chloride (KCl; Nacalai Tesque); 3‐morpholinopropane‐1‐sulfonic acid (MOPS; Nacalai Tesque); potassium hydroxide (KOH; Wako); Citric acid (Wako); Acetic acid (Kishida Chemical Co., Ltd); poly(dT)_50_ (Fasmac Co., Ltd., Kanagawa, Japan); Trypsinized fragments from lysozyme 3 (Tf3; Greiner Bio‐One Co. Ltd, Tokyo, Japan). DPhPC was diluted to 20 mg/mL in *n*‐decane. Buffered electrolyte solutions (1 m KCl, 10 mM MOPS, pH 7.0) were prepared using ultrapure water, which was obtained from a Milli‐Q system (Millipore, Billerica, MA, USA). Wild‐type alpha‐hemolysin (αHL; Sigma–Aldrich, St. Louis, MO, USA) was obtained as the monomer polypeptide, isolated from *Staphylococcus aureus* in the form of a powder and dissolved at a concentration of 1 mg/mL in ultrapure water. For use, samples were diluted to the designated concentration using a buffered electrolyte solution and stored at −80°C. The poly(dT)_50_ was dissolved in ultrapure water and stored at −80°C. Prior to use, it was heated at 98°C for 5 min, slowly cooled to room temperature, and then stored at −20°C. The poly‐L‐lysine hydrobromide (L‐PLL; Mw: 30 000–70 000, Sigma–Aldrich) and short‐poly‐L‐lysine hydrobromide (S‐PLL; Mw: 10 000, Alamanda Polymers, Inc., Huntsville, AL, USA), and Tf3 were dissolved at the designated concentration in ultrapure water.

### 2.2 *E. Coli* Expression and Purification of Epx4

N‐ and C‐terminus of the DNA fragments of Epx4 (459 and 462 nucleotides, respectively) were synthesized (Thermo Fisher Scientific). The codons were optimized to those of *E. coli*. The expression vector of Epx4 was constructed by inserting these two fragments into the multiple cloning site of pET‐26b‐based vector [26] with In‐fusion HD Cloning Kit (Takara Bio Inc.), in which His6‐tag was attached at the C‐terminus.

Epx4 was overexpressed and purified according to the method of Xiong et al. [24], with minor modifications (Text S2). Epx4 was expressed in *E. coli* strain BL21 (DE3) under the control of a T7 promoter. *E. coli* BL21 (DE3) transformed with the expression vector for Epx4 was cultured on LB media supplemented with 25 mg·L‐1 kanamycin at 37°C with shaking at 120 r.p.m. When OD600 reached 0.6, isopropyl β‐D‐thiogalactopyranoside was added at a final concentration of 0.2 mM to induce the expression of Epx4, and the culture was further incubated overnight at 20°C with shaking at 120 r.p.m. Cells were collected by centrifugation at 4000 × *g* for 10 min at 10°C using an Avanti HP‐20 centrifuge (Beckman Coulter, Inc., Pasadena, CA, USA). The collected pellet was resuspended in 70 mL of sonication buffer containing 20 mM Tris‐HCl (pH 7.5), 200 mM NaCl, and 10% (v/v) glycerol, and then sonicated on ice at an output value of 5 and 50 s duty for 10 min with an Ultrasonic Disruptor (Tomy LST‐100; Tomy Seiko Co., Ltd, Tokyo, Japan). The disrupted cells were then centrifuged at 40 000 × *g* for 30 min at 4°C using the Avanti HP‐20 centrifuge. The soluble fraction was filtered using a 0.45 µm filter (Merck, Tokyo, Japan) and loaded onto a 1 mL column of Ni‐Sepharose 6 Fast Flow (Cytiva, Tokyo, Japan). The column was washed with sonication buffer containing 20 mM imidazole, and the bound protein was eluted using a gradient of imidazole in the sonication buffer from 20 mM to 500 mM. Fractions containing Epx4 were filtered using a 0.22 µm filter (Merck) and further purified with HiLoad 16/600 Superdex 75 (Cytiva) gel filtration columns using the buffer composed of 20 mM Tris (pH 7.5), 200 mM NaCl, and 250 mM imidazole. The fractions containing Epx4 were collected and dialyzed overnight into 20 mM Tris, pH 7.5, 200 mM NaCl at 4°C. The purified Epx4 samples were stored at −80°C until use and thawed immediately before channel‐current measurements. Under these storage conditions, pore‐forming activity was confirmed even after approximately one year of storage.

### 2.3 Amplification of the DNA of Epx4 Nanopore for Cell‐Free Synthesis

For cell‐free synthesis, polymerase chain reaction (PCR) was performed to amplify the DNA that contains the T7 promoter and the ribosome binding site (RBS), and the target protein sequence. The pET‐26b plasmids encoding Epx4 have a C‐terminal His6 tag on each open reading frame for purification using cobalt‐based immobilized metal ion affinity chromatography (IMAC) magnetic beads purification. PCR was performed using 25 µL of KOD SYBR qPCR Mix, 1 µL of 1 ng/µL template plasmid, 1 µL of 10 µM primer pair (Table 1), and 22 µL of ultrapure water. The final reaction volume was 50 µL.

**TABLE 1:** Primers.

Forward primer	5’	GAAATTAATACGACTCACTATA	3’
Reverse primer	5’	GTTATGCTAGTTATTGCTCA	3’

PCR was performed using Thermal Cycler Dice Real Time System Lite (Takara Bio Inc.) to amplify the template DNA with the following program: preheating at 98°C for 2 min; 40 cycles of 98°C for 10 s, 58°C for 10 s, 68°C for 1 min (Figure S1a). Then, the DNA product was purified using the NucleoSpin Gel and PCR Clean‐up kit. Purification was performed according to the manufacturer's protocol except for the elution step, in which 30 µL of DNase‐free water (Millipore) was used. The purified sample was stored at 4°C. To confirm the amplified product, we conducted electrophoresis using 1.5% agarose gel in 1 × TAE buffer at a constant voltage of 100 V at room temperature. After electrophoresis, the gel was stained with diluted SYBR Green I solution and visualized under blue LED illumination; images were acquired using an LED transilluminator (Bio Craft Co., Ltd., Tokyo, Japan) (Figure S1b). The concentration of purified DNA was measured by absorbance at 260 nm using a NanoDrop 2000c spectrophotometer (Thermo Fisher Scientific K.K.).

### 2.4 Cell‐Free Synthesis and Purification of Epx4 Nanopore

Purefrex 2.0, a reconstituted cell‐free protein synthesis system, was used for cell‐free synthesis. The system is constructed from purified *E. coli* components necessary for protein synthesis. It contains several factors involved in initiation, elongation, termination, aminoacyl t‐RNA synthetase, and ribosome, among others. Since only the required components are used without cell extracts, the reaction is easily controlled and exhibits high reproducibility [27]. The template DNA contained the T7 promoter sequence, the ribosome binding site, and the target DNA sequence. The template DNA was added to achieve a final concentration of 3 ng/µL. In addition to the template DNA, the reaction solution contains 10 µL of Solution I, 1 µL of Solution II, 2 µL of Solution III, and ultrapure water. The final reaction volume was 20 µL. Protein expression was performed at 37°C for 6 h on a heat block.

### 2.5 His‐Tag Purification

Epx4 monomers were purified using Dynabeads His‐Tag Isolation and Pulldown for cobalt‐based IMAC. Purification was performed according to the protocol provided. First, 10 µL of the beads and ethanol solution was separated using a magnetic stand (MagneSphere Technology Magnetic Separation Stands, Promega Corp., Madison, WI, USA). A 5 µL aliquot of the reaction product was taken in advance for electrophoresis. The remaining 15 µL was mixed with 55 µL of Binding/Wash buffer (300 mM KCl, 20 mM Mg(OAc)2·4H2O, 50 mM Tris‐HCl; pH 8.0) and incubated for 10 min at room temperature. Then, the solution was added to the tube containing the beads and mixed thoroughly. The tube was placed on the magnet for 2 min, and the supernatant was removed. After that, the beads were washed four times with 30 µL of Binding/Wash buffer. Finally, 20 µL of His elution buffer (1 m KCl, 10 mM MOPS, 300 mM imidazole; pH 7.0) was added to the beads, incubated for 5 min at room temperature, placed on the magnet for 2 min, and the supernatant was collected. The elution step was repeated twice, and the eluate was stored at −30°C. The concentration of purified Epx4 was measured by absorbance at 280 nm using a NanoDrop 2000c spectrophotometer.

### 2.6 SDS‐Polyacrylamide Gel Electrophoresis (PAGE)

Electrophoresis was performed to confirm protein expression. Epx4 was mixed with EzApply at a volume ratio of 1:1. This mixture was applied to a p‐PAGEL mini‐slab gel in EzRun solution at a constant current of 20 mA using an AE‐6530P system (ATTO). After electrophoresis, the gel was stained and visualized under blue LED illumination. Images were acquired using an LED transilluminator (Bio Craft Co., Ltd.). To promote oligomerization of the Epx4 monomer, liposomes with a diameter of 100 nm were prepared from DOPC. DOPC lipids were dried to form a lipid film and then rehydrated in 10 mM Tris‐HCl buffer at pH 7.5. The suspension was vortexed and sonicated at 25°C for 60 min. The liposomes were resized by extrusion through a 100 nm polycarbonate membrane 11 times. Finally, 25 µL of Epx4 solution (4.2 µM) was mixed with 40 µL of the DOPC liposome suspension.

### 2.7 Fabrication of Microdevices

The microdevice was designed using Rhino CAM (3DS) and fabricated by machining a 6.0 mm‐thick, 10 × 10 mm PMMA plate using a 3D modeling machine (MM‐100, Modia Systems, Saitama, Japan) (Figure S2a). The separator was cut from 0.2 mm‐thick, 4.0 × 4.5 mm PMMA plate. Two chambers (2.0 mm diameter and 4.5 mm depth) and a chase between the chambers were manufactured on the device body (Figure S2a). Each chamber had a 0.55 mm through‐hole in the bottom and Ag/AgCl electrodes set into this hole (Figure S2a). A polymeric film made of parylene C with a thickness of 5 µm was patterned with a single pore (100 µm diameter) using a conventional photolithography method and then sandwiched between the separators (Figure S2b). The two separators with the polymeric film sandwiched between them were inserted into the chase to separate the chambers.

### 2.8 Lipid Bilayer Preparation and Reconstitution

BLM was formed using the microdevice by the droplet contact method [28, 29, 30, 31, 32, 33, 34] (Figure S2c). In this method, two lipid monolayers contact each other and form a BLM. We first applied 0.3 µL of 20 mg/mL DPhPC dissolved in *n*‐decane to each chamber. The two chambers were separated by a separator, named the recording and ground chambers.

Channel current measurement for pore‐forming evaluation was performed using a Pico patch‐clamp amplifier (Tecella, Foothill Ranch, CA, USA). A volume of 4.7 µL of the buffer solution containing the Epx4 monomer was added to the ground chamber. The buffer solution (4.7 µL) without Epx4 was added to the recording chamber. Within a few minutes of adding the solutions, BLM formed, and Epx4 formed nanopores from the poured chamber by reconstitution in the BLM. If the BLM ruptured during this process, it was recreated by tracing at the interface of the droplet with a hydrophobic stick. The pore conductance was calculated by dividing the step‐signal current value by the applied voltage. The current value used for this conductance calculation was the average current over 1 s after pore opening. A constant voltage of +50, +100, and +150 mV was applied to the recording chamber. Other measurement conditions were a sampling rate of 40 kHz, filtered at about 8 kHz with the built‐in low‐pass Bessel filter. All measurements were performed at room temperature.

### 2.9 Signal Analysis for Pore Forming Evaluation

To evaluate the pore‐forming activity of Epx4, current signals were divided into stable pore‐formation signals and unstable pore‐formation signals by using the signal classification method, which was previously proposed [35]. Based on this method, the current signals were classified into three types: step, square top, and unstable by analyzing the shape of the time‐current traces. The detailed definitions of signal classification are described in Figure S3a–d. Step and square top signals are classified as stable pore‐formation signals. Stable pore‐formation signals are characterized by a rapid current rise (shorter than 10 ms) and the 95% confidence interval of the pore current value is less than 10 times that of the baseline. Signals that maintained a constant current state of more than 1 s were defined as the step signal, and the signal with less than 1 s constant current value was defined as the square top signal. The other signals were classified as unstable signals. For example, the signals that show a sharp signal rise in less than 10 ms, and the 95% confidence interval of the pore current value is more than 10 times that of the baseline, or the signals that show a blunt signal rise in more than 10 ms.

### 2.10 Ion Selectivity

To demonstrate the ion selectivity experimentally, we investigated *P_K_/P_Cl_*, which is the permeability ratio of cations vs. anions given by the Goldman–Hodgkin–Katz (GHK) equation [7]:

$$V_{r}=\frac{\mathrm{RT}}{F}\;\ln(\frac{\sum_{i}^{N}P_{K_{i}^{+}}{\left[K_{i}^{+}\right]}_{\mathrm{trans}}+\sum_{j}^{N}P_{{\mathrm{Cl}}_{j}^{-}}{\left[{\mathrm{Cl}}_{j}^{-}\right]}_{\mathrm{cis}}}{\sum_{i}^{N}P_{K_{i}^{+}}{\left[K_{i}^{+}\right]}_{\mathrm{cis}}+\sum_{j}^{N}P_{{\mathrm{Cl}}_{j}^{-}}{\left[{\mathrm{Cl}}_{j}^{-}\right]}_{\mathrm{trans}}}$$

where *Vr* is the reversal potential, *R* is the universal gas constant, *T* is the temperature, *F* is Faraday's constant, *P* is the permeability of each ion, and [ion] is the ion concentration of each chamber.

### 2.11 Noise Frequency Analysis

To evaluate pore‐derived noise, we calculated the noise frequency. The noise frequency (/s) was defined as the number of blocking signals divided by the open‐state duration (s). Error bars indicate the standard error of the mean (SEM), calculated as the standard deviation of the frequencies obtained from *N* independent nanopores divided by √*N*.

### 2.12 Single‐Molecule Detection

The channel current was monitored using a Pico patch‐clamp amplifier for ssDNA detection and an Axopatch 200B amplifier (Molecular Devices, San Jose, CA, USA) for PLL detection. In ssDNA detection, a constant voltage of +100 mV was applied to the recording chamber. In the ground chamber, Epx4 monomers and the ssDNA were added. In neutral peptide (Tf3) detection [9, 36, 37], a constant voltage of +50 mV was applied to the recording chamber, which contained Tf3 peptides and Epx4 monomer solutions. In these experiments, the sampling rate was 40 kHz, and the low‐pass filter was set to approximately 8 kHz. In PLL detection, a constant voltage of +150 mV was applied to the recording chamber, which contained Epx4 monomers and the PLL. For αHL measurements, the detection experiments were conducted under the same conditions as those for Epx4, including salt concentration, pH, concentration of each target molecule, and experimental equipment. Other measurement conditions included a sampling rate of 100 kHz, low‐pass filtering at approximately 10 kHz with the built‐in Bessel filter, and a gain of 5 GΩ. All measurements were performed at room temperature. Most measurements were performed by the same researcher under consistent experimental conditions, and the reproducibility of the observed trends was confirmed across independent recordings.

### 2.13 MD Simulations

All‐atom molecular dynamics (MD) simulations of Epx4 were performed to investigate pore stability and channel characteristics. The structure of wild‐type Epx4 oligomer was obtained from the Protein Data Bank (PDB ID: 7T4D) [24]. We prepared the starting structure by using CHARMM‐GUI membrane builder [38]. Epx4 oligomer was embedded in the PHPC lipid bilayer. The β‐barrel of Epx4 was embedded in the bilayer while its top, cap, and rim domains protruded above the membrane. K^+^ and Cl^−^ ions were added to neutralize the system, and the final concentration was 1.0 m in the simulation box with initial dimensions ∼12.4 × 12.4 × 27.4 nm^3^. The model was described by the CHARMM36 force field [39]. TIP3P was used as a water model, and NBFIX was used for ions [40, 41]. All MD simulations were run using the GROMACS software [42]. The equilibration followed a quite standard pathway often used in nanopore simulations [43]. In brief, the system was first energy‐minimized using the steepest‐descent algorithm and then equilibrated following the protocol suggested by CHARMM‐GUI. In the first 5 steps, positional restraints on the protein backbone and side chains and on the lipids, as well as dihedral restraints, were gradually reduced while the time step was increased from 1 to 2 fs. In the final equilibration step, only a weak positional restraint on the protein backbone was maintained, and restraints on side chains, lipids, and dihedrals were removed. During several equilibration steps, the pressure was controlled (NPT runs). The system reached stable box dimensions of approximately 12.1 × 12.1 × 26.7 nm^3^ under NPT conditions. Subsequently, production runs were carried out for 90 ns in the NVT ensemble at 298 K, maintained by a Nosé–Hoover thermostat. Constant biased voltages of ±100 and ±200 mV were applied along the *z*‐axis. During the production stage, position restraints were applied to the lipid headgroups to ensure membrane stability. Furthermore, to prevent structural drift and maintain the vertical alignment of the pore, position restraints were imposed on the Met170 and Ala199 residues, located respectively in the upper and central regions of the monomeric chains. The resulting structures were visualized with PyMOL [44] and visual molecular dynamics (VMD) [45]. The equilibrations were run on a computer with an Intel Core i9‐13900F CPU and an NVIDIA GeForce RTX 4070 GPU, while the production runs were run on Leonardo HPC (CINECA). MD simulations of αHL were performed similarly. The ionic and electroosmotic currents were measured using the same protocol adopted in previous reports [43, 46, 47]. Ion density maps were calculated using the VMD Volmap plug‐in, as described by Di Muccio et al. (2022) [46].

### 2.14 Estimation of Surface Charge Inside Epx4 in Different pH Conditions

Epx4 at different pH levels was processed using PDB2PQR [48]. The pKa values of side chains were estimated using PROPKA3 [49, 50]. Electrostatic maps were then calculated using APBS [51] at the appropriate ion concentration and loaded in PyMOL for visualization [44].

### 2.15 Data Analysis and Statistical Analysis

The recorded data from the Pico patch‐clamp amplifier and the Axopatch 200B were acquired with Clampex 9.0 software (Molecular Devices) through either a Digidata 1440A or a Digidata 1550B analog‐to‐digital converter (Molecular Devices). The current data were analyzed using Clampfit 11.3 (Molecular Devices), Excel (Microsoft Corp., Redmond, WA, USA), and OriginPro 2022b (OriginLab Corporation, Northampton, MA, USA). Capture frequency (/s) was calculated by dividing the number of blocking signals by the open‐state duration time (s). Error bars indicate the standard error of the mean (SEM), calculated as the standard deviation of the frequency calculated on *N* independent nanopores divided by the √*N*. For a fair comparison, we analyzed the event frequency in αHL using the same analysis time as that used for Epx4 when comparing the event frequency of ssDNA and PLL. To investigate the statistical differences, a two‐sided Welch's *t*‐test was used, whereas a two‐sided Mann‐Whitney *U*‐test was used when normality could not be assumed. The statistical analysis was conducted using OriginPro 2022b.

The machine learning process consisted of three parts: data collection, feature extraction, and model training. The machine learning analyses were performed using Python 3.12. The sample sizes for S‐PLL (N >3, n = 169) and L‐PLL (N > 3, n = 120) were matched for Epx4 and αHL, respectively. *N* indicates the number of independent nanopores, and *n* indicates the number of blocking signals. After removing pore‐derived noise, blocking signals of S‐PLL and L‐PLL through Epx4 nanopore were collected to form a labeled dataset. The label for each event was assigned according to the PLL type used for data generation. The event features were as follows: mean blocking ratio, log_10_(duration time), Std, kurtosis, skewness, and number of KDE peaks. The event features were extracted from each blocking signal. The collected event features were then split into training (80%) and testing (20%) sets using stratified sampling to preserve class balance. Multiple classifiers implemented in scikit‐learn were evaluated, including Decision Trees, Random Forest, Support Vector Machine (SVM), Neural Network, Naïve Bayes, and Discriminant Analysis. Bayesian optimization was conducted for hyperparameter tuning. To ensure robustness, training and testing were repeated under multiple random seeds to ensure reproducibility. Stratified k‐fold cross‐validation was conducted to prevent overfitting. Stratification was used to preserve the class balance in each fold. After hyperparameter tuning, probability calibration was conducted to align the predicted class probabilities with the observed class frequencies. Since the sample sizes for S‐PLL and L‐PLL differed, we applied balanced sample weights to correct for this disparity. We then selected the threshold that maximized the F1 macro score for L‐PLL and used this value for subsequent external validation. A confusion matrix was generated based on the results of the classification model.

## 3 Results and Discussion

### 3.1 Confirmation of the Number of Constrictions in Epx4

A previous study revealed the crystal structure of Epx4, which consists of four domains: top, cap, rim, and stem domains [24] (Figure 1a). Unlike existing natural nanopores, such as αHL [1], AeL [52], MspA [53], CsgG [54], or FraC [55], Epx4 has an extra β‐barrel referred to as the top domain. The cap, rim, and stem domains of the Epx4 protomer are structurally similar to the protomers of αHL [24], which is one of the most widely used nanopores for nucleotide detection (Figure 1b). The sensitivity of nanopore sensing depends on the pore size, the internal geometry, and the chemical environment. To investigate and visualize the basic geometry inside Epx4, we performed a POVME3.0 analysis [56] following the protocol described in Reccia et al. [57]. This approach allowed for a precise volumetric mapping of the internal cavity by defining the lumen length through a grid‐based inclusion sphere. The analysis indicated that Epx4 has several potential constrictions located at E43, K57, E155, and E171. According to the equivalent radius profile, the narrowest regions are positioned within the top domain at E43 and at the bottom of the transmembrane β‐barrel at E171, both exhibiting an equivalent radius of approximately 10 Å. Residue K57 defines a slightly wider constriction with an equivalent radius of approximately 11–12 Å (Figure 1c). For comparison, POVME analysis was also performed on αHL to examine its internal structure (Figure 1d). Although previous studies have reported structural similarities between the αHL and the Epx4 protomer [24], our POVME‐based volumetric profiles revealed differences in their internal geometries, particularly in the locations of the constrictions (Figure 1c,d). In αHL, the major constriction is located at the top of the transmembrane region, while Epx4 has constrictions not only at the bottom of the transmembrane region (E171) but also at the extramembrane top domain (E43, K57). The diameters at E43 and K57 were 2.0 and 2.3 nm, respectively. These structural properties suggest that Epx4 may provide detailed blocking‐signal information during single‐molecule detection due to enhanced interactions within its multi‐constriction structure.

### 3.2 Expression and Functional Characterization of Epx4 Nanopores

**FIGURE 2:**
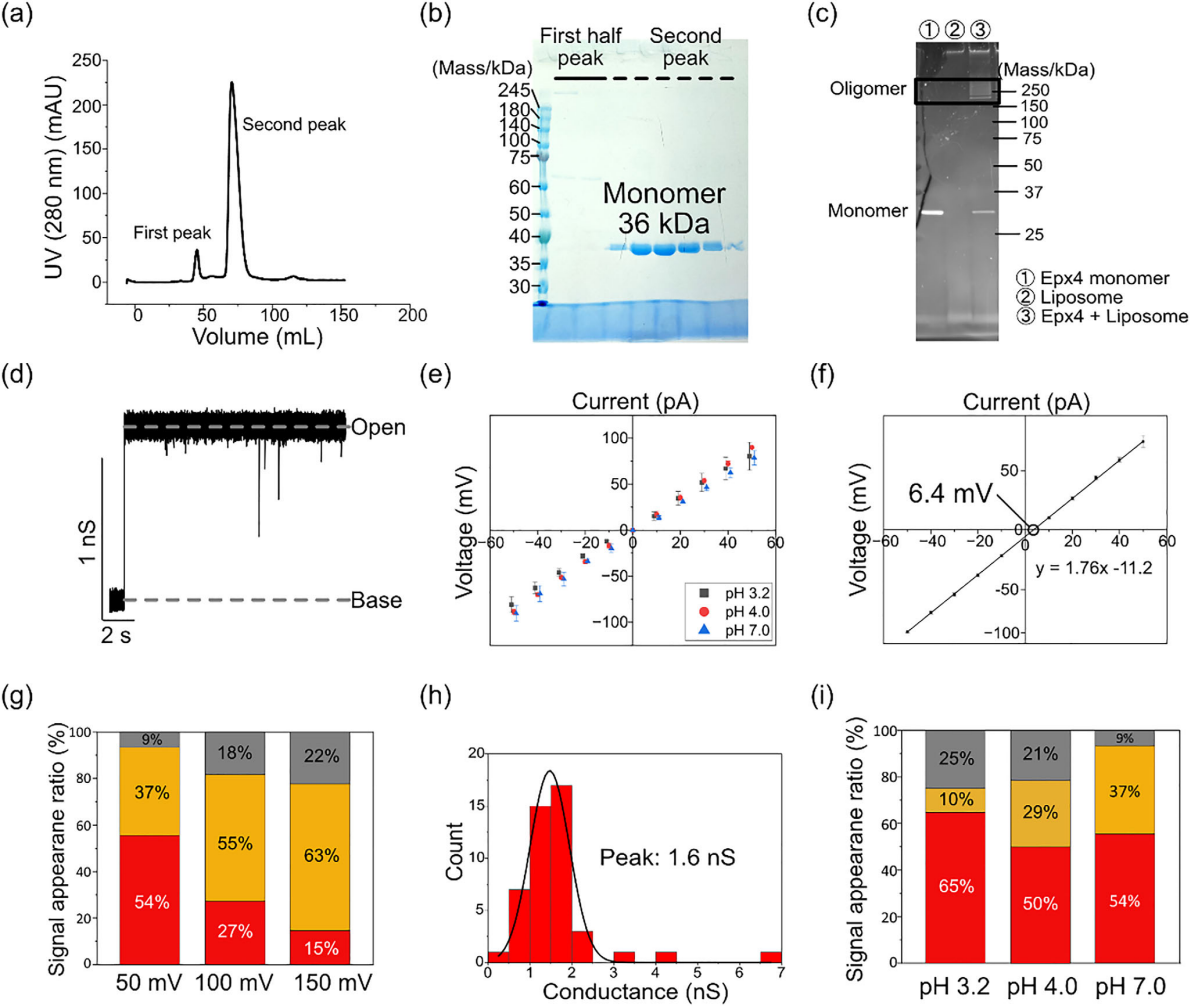
Expression and characterization of Epx4 nanopore. (a) Size exclusion chromatography profile of Epx4 after *E. coli* expression. The absorbance at 280 nm is plotted against elution volume. (b) SDS‐PAGE of eluted fractions to confirm the *E. coli* expression and purification. (c) SDS‐PAGE to confirm the cell‐free synthesis and His‐tag purification with and without the incubation of liposomes. (d) Typical current‐time traces of Epx4, which is classified into step signals. (e) Single‐channel *I–V* curve of Epx4 in 1 m KCl at pH 7.0, 4.0, and 3.2. The error bars of the data are based on three independent nanopores. (f) Measurement of ion selectivity of Epx4. Current through Epx4 as a function of voltage in the presence of 1 m/0.1 m KCl concentration at pH 7.0. The data represent the mean ± SEM. (g) Results of signal classification of Epx4 at +50 mV (*N* = 46), +100 mV (*N* = 77), and +150 mV (*N* = 27). Step (red), square top (orange) mean stable signals; the other signals (gray) are unstable. Signal appearance ratio was calculated by dividing the number of signals classified into each category based on determined signal classification criteria by the total number of signals. (h) The conductance distribution of the step signals in 1 m KCl at pH 7.0 (*N* = 46). The histograms were fitted to a Gaussian distribution. The peak was 1.6 nS. (i) Comparison of signal classification at the voltage of +50 mV among pH 3.2 (*N* = 20), 4.0 (*N* = 14), and 7.0 (*N* = 46). The definition of color is the same as in (g). The channel current measurements at pH 3.2 were conducted in 1 m KCl, 10 mM citric acid; those at pH 4.0 were conducted in 1 m KCl, 10 mM acetic acid.

We next expressed the protein using both *E. coli* expression and cell‐free synthesis. Size exclusion chromatography (SEC) of the *E. coli*‐expressed sample showed a single peak corresponding to the Epx4 monomer (∼36 kDa; Figure 2a), which was also confirmed by SDS‐PAGE (Figure 2b). In the cell‐free synthesis samples, we observed a single band indicating the Epx4 monomer, but no oligomeric species were detected by the SDS‐PAGE analysis (Figure 2c). When the Epx4 monomers were incubated with DOPC liposomes for 1 h, bands corresponding to the oligomers appeared (Figure 2c), consistent with previous research [24].

To evaluate the pore‐forming properties of Epx4 in a BLM, we conducted channel‐current measurements using a microdevice. The BLM was prepared by the droplet contact method [28, 29, 30, 31, 32, 33, 34], and the current signals of Epx4 were analyzed. After BLM formation, an increase in the current signal was observed, indicating the formation of an Epx4 nanopore (Figure 2d). The *I–V* curve exhibited a linear slope, suggesting that the structural asymmetry of Epx4 did not result in detectable rectification under the present conditions (Figure 2e). To evaluate ion selectivity, we measured the current under asymmetric KCl concentrations on either side of the nanopore (1 M vs. 0.1 m). The slopes of the linear fits to the *I*–*V* curve were 1.76 and 1.74 for ΔV >0 and ΔV< 0, respectively, indicating no rectification under these conditions (Figure 2f). We quantified the ion selectivity using *P_K_/P_Cl_*, the permeability ratio of cations to anions, calculated based on the Goldman–Hodgkin–Katz (GHK) equation [7] (see Methods). At pH 7.0, the reversal potential *Vr* was 6.4 mV, corresponding to a *P_K_/P_Cl_* value of 1.4 (Figure 2f). These results indicate that Epx4 is a slightly cation‐selective nanopore under the present experimental conditions. To investigate whether Epx4 has single‐molecule sensing capabilities, we classified the open‐current signals using the classification method that we previously proposed [35]. The current signals were classified into three types: step, square top, and unstable signals, the latter representing all other irregular or short‐lived signals that did not meet the criteria of the first two categories. The step signal, representing stable pore formation lasting longer than 1 s, is the most suitable for single‐molecule detection because of its high signal‐to‐noise ratio. In contrast, the square top signals reflect gating behavior, where the conductance drops after pore formation and remains in a closed or partially closed state for several seconds, preventing a stable open‐pore condition. Signals with an open‐pore duration shorter than 1 s were classified as square top (Figure S3a–d). Accordingly, step and square top signals were treated as stable events, while unstable signals were excluded. At both +150 mV and +100 mV, approximately 80% of the events were classified as stable signals (Figure 2g), with square top signals alone accounting for more than 50% (Figure 2g). To improve the stability of the open‐pore state, we reduced the applied voltage because gating events were frequent at higher voltages [58]. At +50 mV, the ratio of step signals increased to 54%, indicating that the gating behavior of Epx4 depends on the applied voltage (Figure 2g). Under this condition, Epx4 formed stable open pores with durations on the order of 10 s at submicromolar concentrations, providing a sufficient time window for subsequent single‐molecule sensing experiments (Figure S3e). The conductance histogram showed a single peak of 1.6 nS in 1 m KCl, and the conductance was nearly linear as a function of KCl concentration (Figure 2h and Table S3 and Figure S4a). This value is higher than that of αHL (∼1 nS) [59], likely due to the larger pore radius of Epx4 (Figure 1c,d). Increasing ionic strength from 1 m to 3 m KCl did not reduce gating, as the fraction of square top signals remained at ∼55% (Figure S4b). Salt‐dependent gating behavior has been reported for γ‐hemolysin [60], but such dependence was not observed for Epx4 under the present conditions. Because protonation of charged residues in the pore lumen may affect channel stability and ion transport, we further examined the pore properties under different pH conditions. The pKa values of side chains were estimated using PROPKA [49, 50] (Table S2). At pH 4.0, the surface charge inside Epx4 was dramatically changed. E171 at the narrowest constriction was not completely protonated (Figure S5a and Table S2). The *I–V* curves at pH 3.2, 4.0, and 7.0 are shown in Figure 2e. The *I–V* relationships remained linear and showed no rectification, similar to those at pH 7.0 (Figure 2e). In the signal characteristics analysis, the appearance ratio of unstable signals increased from 9% at pH 7.0 to more than 20% at pH 3.2 and 4.0, while the ratio of step signals was over 50% under all conditions (Figure 2i). The conductance was comparable across conditions (1.6 ± 0.2 (*N* = 7) at pH 4.0, and 1.5 ± 0.2 (*N* = 13) at pH 3.2), and the transient noise frequency showed no significant difference among pH conditions (Figure S5b).

### 3.3 Single‐Molecule Detection of ssDNA and Polypeptide by Using Epx4 Nanopore

**FIGURE 3:**
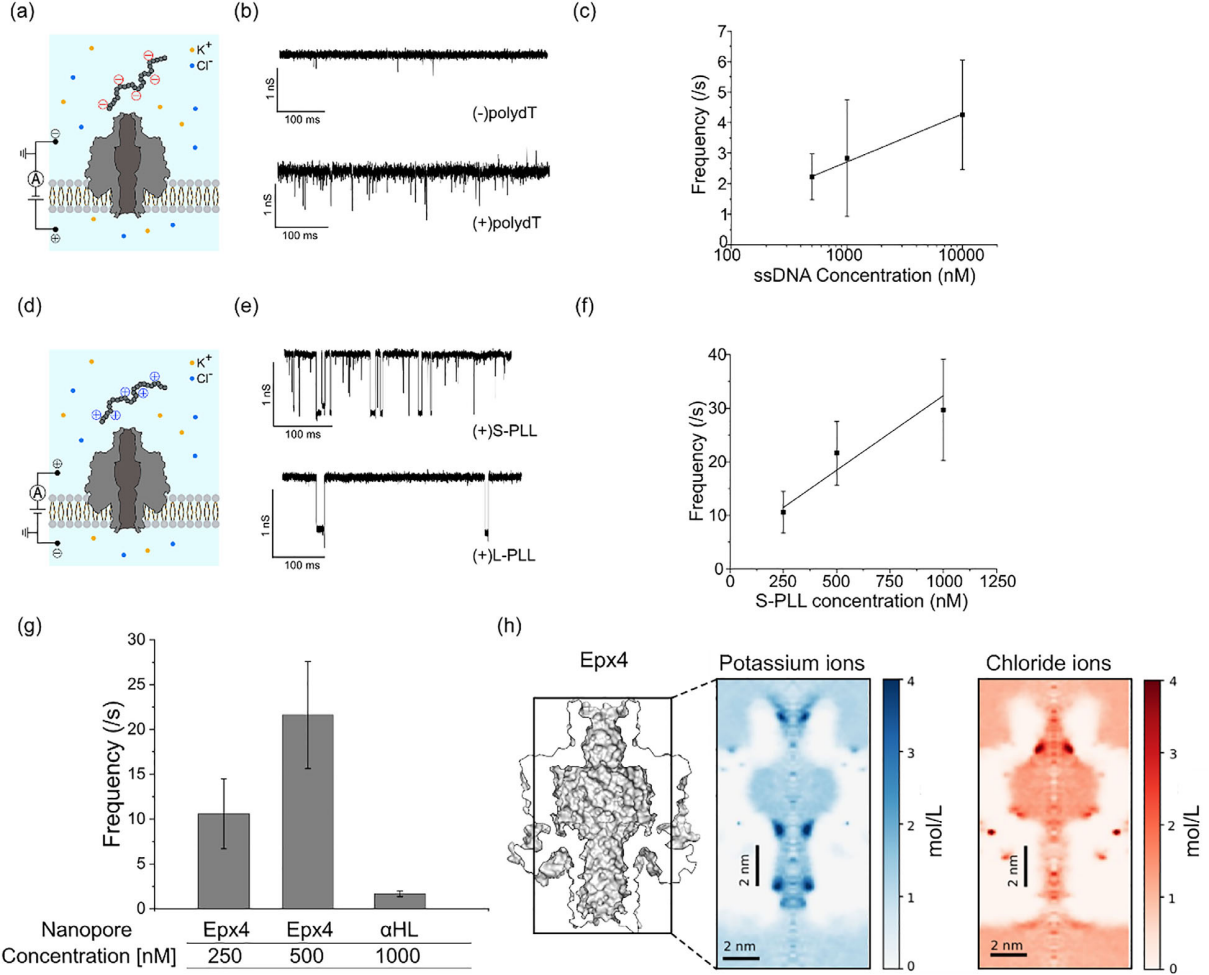
Single‐molecule detection of ssDNA and polypeptide using Epx4 nanopore. (a) Schematic diagram of ssDNA detection through Epx4 nanopore. (b) Typical current‐time traces with polydT_50_ using Epx4 nanopore. The data were obtained in 1.0 m KCl, 10 mM MOPS, pH 7.0, with or without polydT_50_. (c) Event frequency of polydT_50_ as a function of concentration. Data are presented as mean ± SEM from independent nanopores (*N* = 4 for 500 nM, *N* = 4 for 1000 nM, and *N* = 4 for 10000 nM). (d) Schematic diagram of PLLs detection through Epx4 nanopore. The dotted line serves to guide the eye. (e) Typical current‐time traces with PLLs using Epx4 nanopore. The data were obtained in 1.0 m KCl, 10 mM MOPS, pH 7.0, with or without PLLs. (f) Event frequency of S‐PLL as a function of concentration. Data are presented as mean ± SEM from independent nanopores (*N* = 3 for 250 nM, *N* = 3 for 500 nM, and *N* = 5 for 1000 nM). The dotted line serves to guide the eye. (g) Comparison of the S‐PLL event frequency between Epx4 and αHL at different concentrations. (h) Charge density maps at equilibrium, ∆*V* = 0, for 1.0 m KCl, showing the distribution of cations (blue) and anions (red) within the lumen of Epx4. Cations and anions accumulate near residues of opposite charge. The ion density maps were obtained from MD simulation analysis.

Next, we evaluated the sensing capabilities of Epx4 using ssDNA and two types of polypeptides, poly‐L‐lysine (PLL): S‐PLL and L‐PLL, each in separate experiments. For ssDNA measurements, ssDNA was added to the ground chamber to enable electrophoretic capture, with the top domain of Epx4 oriented toward the ground side (Figure 3a). When we added ssDNA, blocking signals were observed (Figure 3b). The frequency of the blocking signals increased as the ssDNA concentration increased, indicating that Epx4 can detect ssDNA (Figure 3c and Figure S6a). Although Epx4 detection frequency was much higher than that of αHL, no statistically significant difference was observed between the two (Figure S6c). For PLL measurements, S‐PLL and L‐PLL were each tested in separate experiments and added to the recording chamber to enable electrophoretic capture, with the top domain oriented toward the recording side (Figure 3d). The frequency of blocking signals increased with PLL concentration, suggesting that Epx4 can detect cationic polypeptides as a nanopore sensor (Figure 3e,f, and Figure S6b). We next compared the polypeptide detection capabilities of Epx4 with those of αHL. For S‐PLL detection, Epx4 showed a higher event frequency than αHL. The duration times were not statistically different (Figure 3g and Table S1). In Epx4, the frequency reached 21.6 ± 6.0 s^−1^ at 500 nM S‐PLL, whereas in αHL the frequency remained 1.68 ± 0.26 s^−1^ even at 1000 nM S‐PLL. When the PLLs were introduced from the *trans* side of Epx4, the open signals were too unstable for quantitative analysis (Figure S7). As reported in αHL, signal frequency and signal shape may change depending on the direction of *cis* or *trans*. Surface charge, pore orientation in the membrane, and the presence of the vestibule may be factors [61, 62, 63, 64].

In nanopore systems, capture frequency is determined by multiple factors, including electrophoresis, electroosmosis, and energy barriers at the pore entrance [65]. In the present system, dielectrophoresis is expected to be negligible (molecules are homogeneously charged), and the dominant effect is electrophoresis. Two additional factors may contribute to the observed difference in event frequency between Epx4 and αHL. The first is the surface charge at the pore entrance. Epx4 has a predominantly negative surface charge, particularly near the entrance, as supported by the accumulation of potassium ions in MD simulations (Figure 3h), whereas αHL has a more positive lumen [43, 66]. This difference may enhance the capture of cationic PLLs in Epx4. A second factor is electroosmotic flow (EOF) (Figure S8a) [67, 68, 69]. MD simulations showed that the predicted EOF direction in Epx4 was consistent with the observed direction of PLL transport (Figure S8b,c). This interpretation is supported by ion selectivity (Figure 2f) and the neutral peptide experiments, where blocking signals were observed when peptide transport occurred in the same direction as the EOF (Figure S9). These findings suggest that EOF may contribute to PLL transport in Epx4, as reported for other nanopores [6, 7, 68, 70].

### 3.4 Comparison of the Polypeptide Identification Capability Between Epx4 and αHL by Using Machine Learning

**FIGURE 4:**
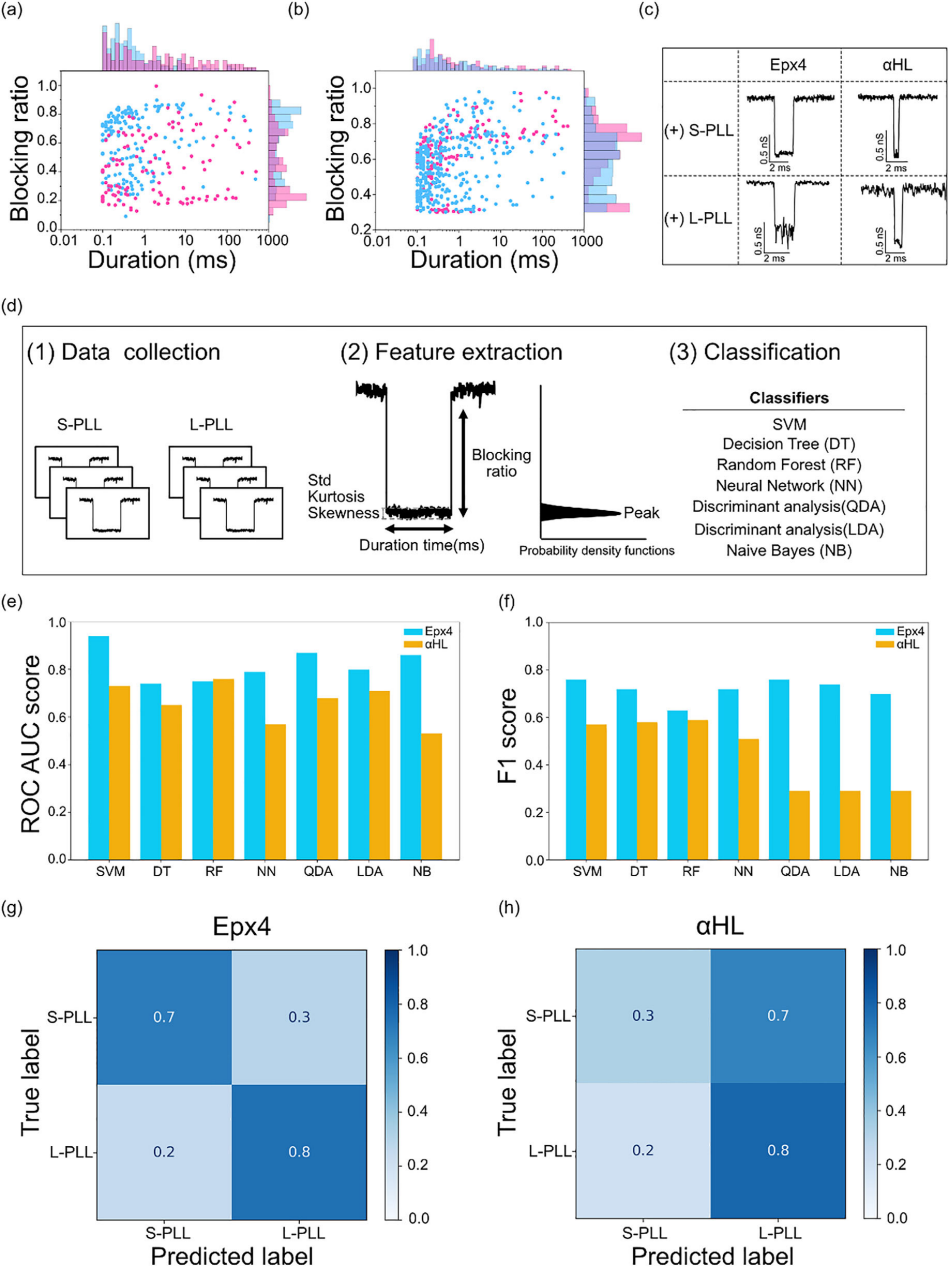
Comparison of the PLL identification ability between Epx4 and αHL by using machine learning. (a) Scatter plot of duration time vs. blocking for Epx4. S‐PLL is colored blue, and L‐PLL is colored pink. (b) Scatter plot of duration time vs. blocking for αHL. S‐PLL and L‐PLL are colored in the same way. (c) Comparison of signal shapes of PLLs between Epx4 and αHL. (d) Illustration of the machine learning methods. The methods consist of three phases: data collection, feature extraction, and classification. (e) ROC AUC score of Epx4 (blue) and αHL (orange). (f) Averaged F1 macro score of Epx4 (blue) and αHL (orange). (g,h) Confusion matrix of Epx4 (g) and αHL (h) of NN models.

Previous research on the CsgG‐CsgF system showed that machine‐learning‐based analysis can extract more information from current signals [19]. Based on this approach, we applied machine‐learning classification methods to compare the polypeptide identification capability between Epx4 and αHL. Figure 4a,b shows scatter plots of duration time vs. current blocking ratio for PLL events. In both Epx4 and αHL, a broad distribution was observed, ranging from transient signals to long and deep blockings, reflecting diverse interactions between PLLs and the nanopore. These overlapping trends suggested that distinguishing PLLs solely from the scatter plots would be difficult. Therefore, we applied machine learning to classify PLL types by utilizing multidimensional features extracted from individual blocking signals.

Previous studies have shown that the signal shapes change depending on the locations of the target molecule that are temporarily trapped or interact within a nanopore. For example, substate blocking signals have been reported in αHL when ssDNA is electrophoretically driven through the pore. Butler et al. attributed these multistate signals to different translocation configurations, modeling the substates as a Mid (ssDNA in the vestibule) and Deep (ssDNA through the constriction) conductance states [71]. Similarly, it has been reported that the signal characteristics of hairpin‐DNA signals depend on whether unzipping occurs inside the vestibule or outside the *cis* entrance [72]. Consistent with these observations, ion density maps from our MD simulations showed localization of potassium ions at the cis entrance and within the β‐barrel (Figure 3h), suggesting that spatially distinct interactions may influence signal behavior. Based on this, we focused on signal shape features. Epx4 exhibited signal characteristics distinct from those of αHL, particularly in blocking signal fluctuation (Figure 4c). To automatically evaluate these signal differences, we performed machine‐learning classification using six features [15, 73]: blocking ratio, duration time, standard deviation (Std), skewness, kurtosis, and number of peaks (Figure 4d and Figure S10). Std and the number of peaks reflect amplitude‐related characteristics of each signal. For Epx4, the ROC AUC score across classification models was 0.82 ± 0.072 (mean ± SD), while for αHL it was 0.69 ± 0.047 (mean ± SD), indicating that Epx4 achieved higher ROC AUC scores in most classification models (Figure 4e). The F1 macro score, which is used to evaluate classification performance, especially when the classes are imbalanced, was 0.72 ± 0.045 for Epx4 and 0.45 ± 0.15 for αHL, showing higher classification performance for Epx4 under the present experimental conditions (Figure 4f). Similar results were obtained when each classifier was trained and predicted three times with different random seeds (Figure S11). The confusion matrices further supported this trend (Figure 4g,h), with classification accuracies of 0.8 for L‐PLL and 0.7 for S‐PLL in Epx4, both higher than those for αHL. While the absolute accuracy is moderate, it is notable that improved classification was achieved even for homopolymeric PLLs, which offer limited chemical diversity. This result suggests that the presence of multiple constrictions in Epx4 enhances the extraction of informative signal features, enabling discrimination based on subtle differences in translocation dynamics.

## 4 Conclusion

This study demonstrated that Epx4 can be applied to a nanopore sensor. Epx4 formed stable pores with predominantly stable events at +100 mV. Lowering the voltage to +50 mV increased the step signals available for single‐molecule detection, improving the step signal appearance ratio from 27% to 54%. The concentration dependence of ssDNA and cationic PLLs indicated that Epx4 can detect at the single‐molecule level. Epx4 showed a higher S‐PLL event frequency (21.6 ± 6.0 s^−^^1^ at 500 nM) than αHL. Although Epx4 produced diverse blocking signals in terms of current blocking ratio and duration, machine‐learning‐based classification showed higher performance compared to αHL. This indicates that Epx4 signals contain features that enable discrimination between S‐PLL and L‐PLL. These features are not fully captured by 2D scatter plots but can be effectively utilized by machine‐learning approaches.

These results support the potential of Epx4 for nanopore‐based protein sensing. Previous studies have shown that mutations in nanopore proteins strongly affect sensing properties (e.g., amino acid type [36, 74, 75], binding‐enhancing mutations [4, 15, 17], and mutation sites [76]). Because Epx4 contains multiple constrictions, it provides a platform in which signal generation can be tuned through mutational design. The present results suggest that these constrictions contribute to the generation of informative signal features. Therefore, introducing mutations at selected constriction sites may enable control of analyte–pore interactions and further improve discrimination performance.

## Conflicts of Interest

The authors declare no conflicts of interest.

## Supporting information




**Supporting File**: smtd70762‐sup‐0001‐SuppMat.docx.

## Data Availability

The data that support the findings of this study are available from the corresponding author upon reasonable request.
