# Supplementary material for: Epx4 Nanopore With Multiple Constrictions for Single‐Molecule Identification
Source: Small Methods. 2026 Jun 11;10(13):e70762. doi: 10.1002/smtd.70762 (PMC13353876; doi:10.1002/smtd.70762)
Supplement: Supplementary file 1 — Supporting File: smtd70762‐sup‐0001‐SuppMat.docx. [file SMTD-10-e70762-s001.docx]

**Epx4 nanopore with multiple constrictions for single-molecule identification**

Ayako Ijuin^1^, Kota Naito^2^, Mana Sato^1^, Virginia Di Toro Mammarella^3^, Nanami Takeuchi^1^, Mauro Chinappi^3^, Yoshikazu Tanaka^2^, and Ryuji Kawano^1^*

1 Department of Biotechnology and Life Science, Tokyo University of Agriculture and Technology (TUAT), 2-24-16 Nakacho Koganei-shi Tokyo 184-8588, Japan

2 Graduate School of Life Sciences, Tohoku University, 2-1-1 Katahira Aoba-ku Sendai Miyagi 980-8577, Japan

3 Department of Industrial Engineering, University of Rome Tor Vergata, Via del Politecnico 1, 00133 Roma, Italy

*rjkawano@cc.tuat.ac.jp

**Table of Contents**

SI Text 1. Current understanding of Epx4

SI Text 2. Protein sequence of Epx4

Table S1. Duration time in S-PLL detection between Epx4 and αHL

Table S2. pKa values of each constriction

Table S3. Single-channel conductance in different KCl concentrations

Figure S1. Confirmation of DNA amplification for cell-free synthesis

Figure S2. Diagram of a microdevice and the principle of the droplet contact method

Figure S3. Detailed criteria for signal classification and long-term stability analysis

Figure S4. Pore properties of Epx4 in different KCl concentrations

Figure S5. Pore properties of Epx4 nanopore in different pH conditions

Figure S6. Event frequency of polydT50 and S-PLL capture

Figure S7. S-PLL detection from the trans side of Epx4.

Figure S8. Ion current and electroosmotic flow from MD simulation

Figure S9. Neutral peptide detection by using Epx4 nanopore

Figure S10. Distribution of each feature for PLLs

Figure S11. Classifier performance across three times

**SI Text 1. Current understanding of Epx4**

Enterococcus pore-forming toxins (Epxs), including Epx4, were reported by Xiong et al (1). They observed Epx1 and Epx2 as a potential small β-barrel pore-forming toxin genes in an *E. faecalis* strain. As additional homologs, their search identified Epx3-8. The known properties of Epx4 are summarized below.

**Structure:** Xiong et al. obtained crystal structures of Epx4, showing that Epx4 assembles into an octameric pore in the presence of 2-methyl-2,4-pentanediol (MPD). The overall structure is similar to α-hemolysin (αHL), comprising cap, rim, and stem domains. A unique feature of Epx4 is the presence of a second β-barrel on the cap domain, referred to as “top domain”.

**Biological role:** Epx2 and Epx3 were shown to recognize the human leukocyte antigen class I (HLA-I) complex, but Epx4 showed no sensitivity. The study further reported that Epx4 did not show high toxicity in the tested cell lines, likely because these cells lack the corresponding receptors. Thus, while the biological roles of Epx2 and Epx3 have been partially elucidated, the specific biological role of Epx4 remains unclear, including its physiological receptor and target cell types.

**Pore-forming properties:** Incubation of Epx1-4 with liposomes induced lysis of artificial membranes *in vitro*. In Epx4, charged residues within the top domain were found to contribute to inter-protomer interactions. Mutations (K51E/K57E) in the top domain reduced the formation of SDS-resistant oligomers and decreased toxicity in HeLa cells without altering the overall structure. Sequence analysis also suggests that the top domain corresponds to the N-terminal latch domain, which is implicated in inter-protomer interactions in other Hla family members.

**Prior electrophysiological or biophysical studies:** To date, channel current measurements have been reported only for Epx1, not for Epx4. Epx1 exhibited no rectification, and its pore diameter was estimated to be ~2.1 nm based on Hille’s equations (2).

**SI Text 2. Protein sequence of Epx4**

SEDNIIGTTTQEIDEHGNVKTIITVKNQQIESYTSTDSGTAKNRSTLTVNANFLNDKYSNELTTILSLNGFIPSGRKFIFPKNNTLKGEMLWPQRYSTAVYNIPLDKSVKITNSTPDNTIRSKEVSNSITYGIGGGIKMEGKQPGANLDANAAITKTISYQQPDYETAKTTSTVTGVNWNTNFTETRDGYTRNSWNPVYGNQMFMYGRYTSNIRNNFTPDYQLSSLITSGFSPSYGLVLRAPKDVKKSRIKVVFARRSETYQQNWDGLNWWGRNFYDTKNPDSLSKVTLTFELDWQNHRVTFIELEHHHHHH

| **Table S1. Duration time in S-PLL detection between Epx4 and αHL** | |
| --- | --- |
|  | **Median** |
| **Epx4** | **0.420 ms** |
| **αHL** | **0.380 ms** |

| **Table S2. pKa values of each constriction** | | | | |
| --- | --- | --- | --- | --- |
| Residue | E43 | K57 | E155 | E171 |
| pKa* | 3.96 | 9.94 | 4.96 | 3.71 |

*Average pKa value, because the pKa values differed slightly among monomers.

| **Table S3. Single-channel conductance in different KCl concentrations** | | | | |
| --- | --- | --- | --- | --- |
| KCl (M) | 0.1 | 1 | 2 | 3 |
| Conductance (nS) | $0.37 \pm0.11$ | $1.50 \pm0.15$ | $2.43 \pm0.67$ | $3.69 \pm0.27$ |


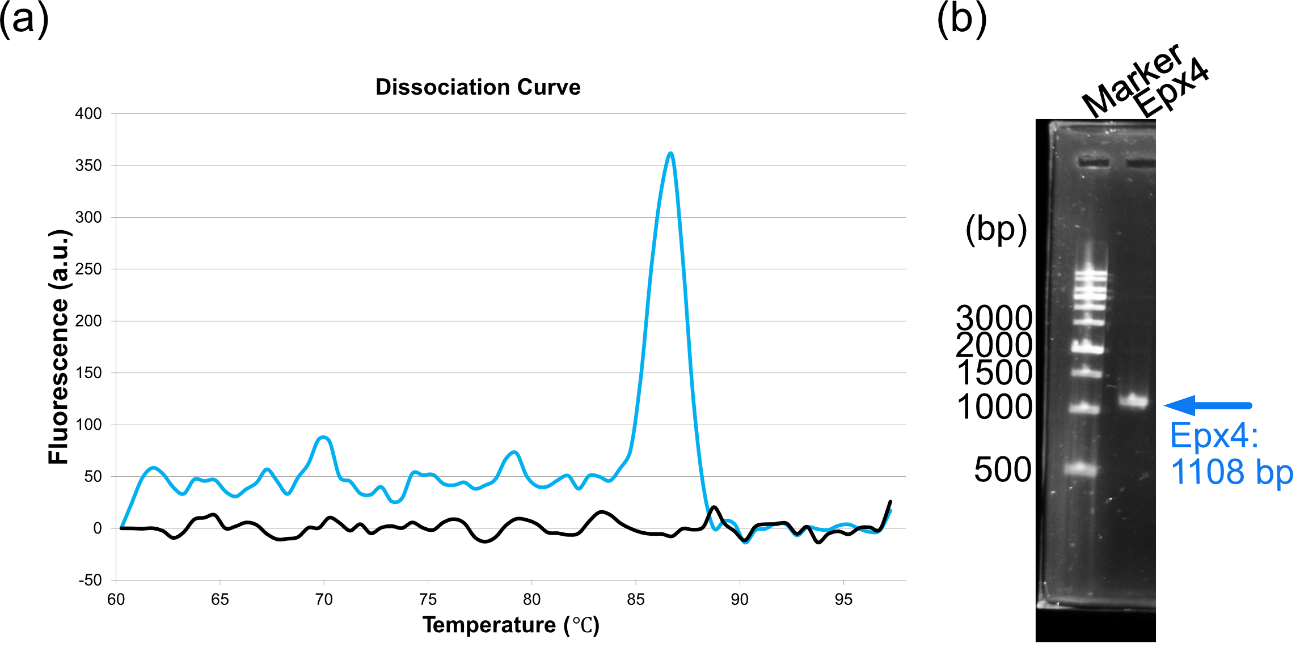


**Figure S1. Confirmation of DNA amplification for cell-free synthesis.** (a) Dissociation curve of PCR. The T_m_ value of the target sequence is 86.1 ℃. (b) An image of an agarose gel after electrophoresis.


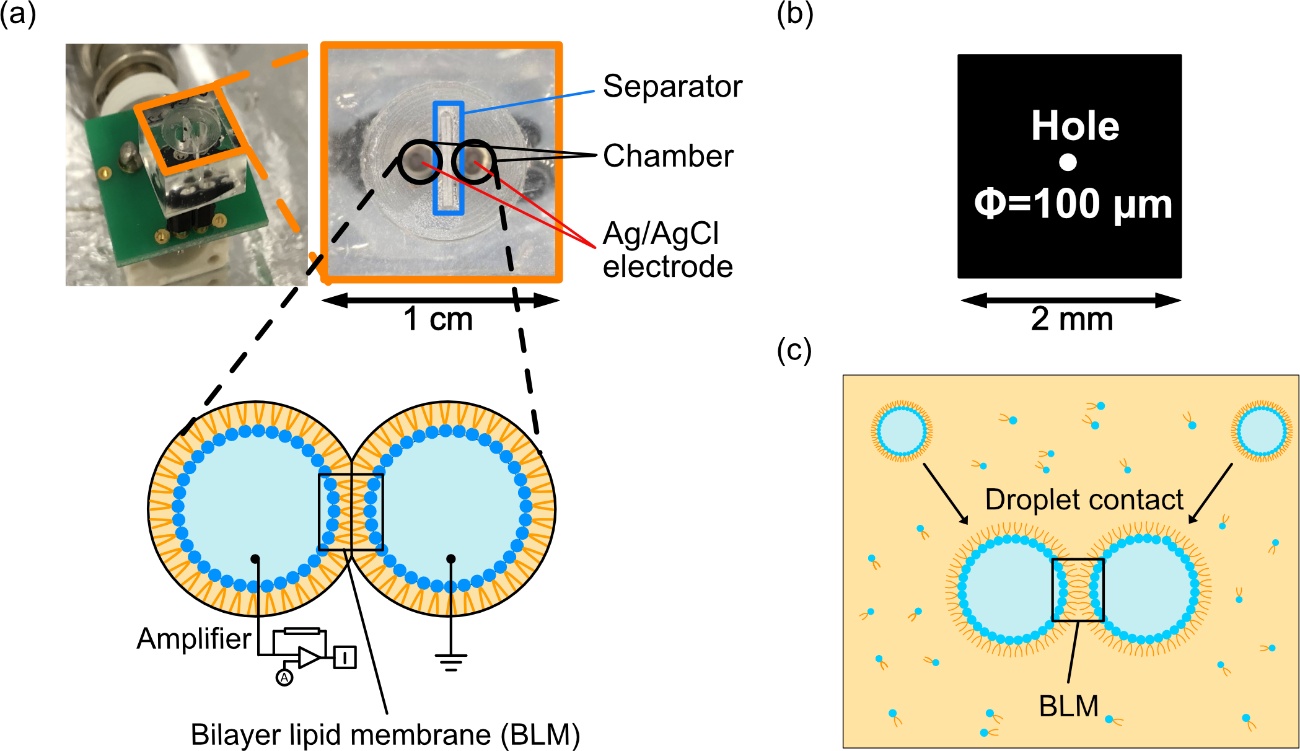


**Figure S2. Diagram of a microdevice and the principle of the droplet contact method.** (a) Microdevice consisting of a separator, chambers, and Ag/AgCl electrodes. When two lipid monolayers contact each other via a parylene film, the BLM is formed. (b) Image of a parylene film, which is sandwiched between the separators. (c) The principle of the droplet contact method.


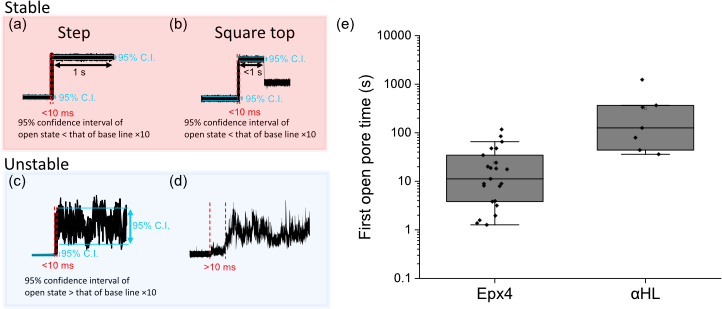


**Figure S3. Detailed criteria for signal classification and long-term stability analysis**. Step (a) and square top (b) are classified as stable pore formation signals. The difference between them is the signal duration (whether it lasts longer or shorter than 1 s). The other signals are classified as unstable signals (c, d). (e) Comparison of the initial open-pore time of Epx4 (*N*=23) and αHL (*N*=7) at 50 mV. The concentration of Epx4 was submicromolar, whereas that of αHL was 5–10 nM. *N* indicates the number of independent nanopores.


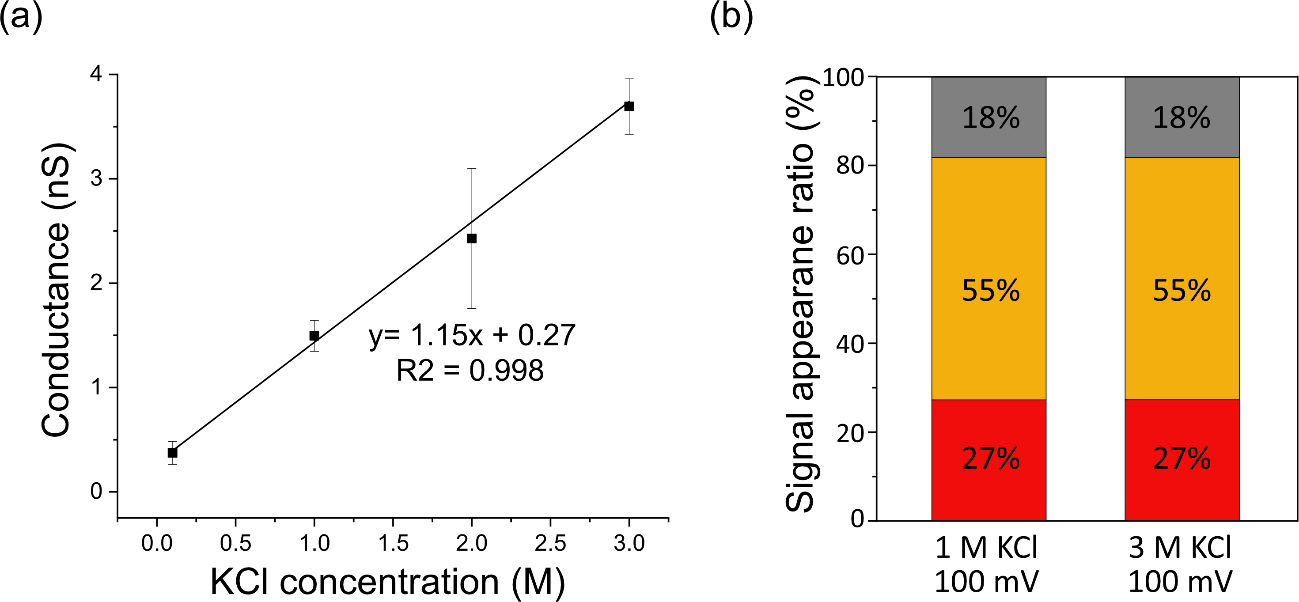


**Figure S4. Pore properties of Epx4 in different KCl concentrations.** (a) Conductance in different KCl concentrations on the original scale. The data represent the mean ± SEM of at least three independent nanopores. (b) Comparison of signal classification in 1 M and 3 M KCl. Step (red), square top (orange) indicate stable signals, the other signals (gray) are unstable signals. Signal appearance ratio was calculated by dividing the number of signals classified into each category based on the determined signal classification criteria by the total number of signals.


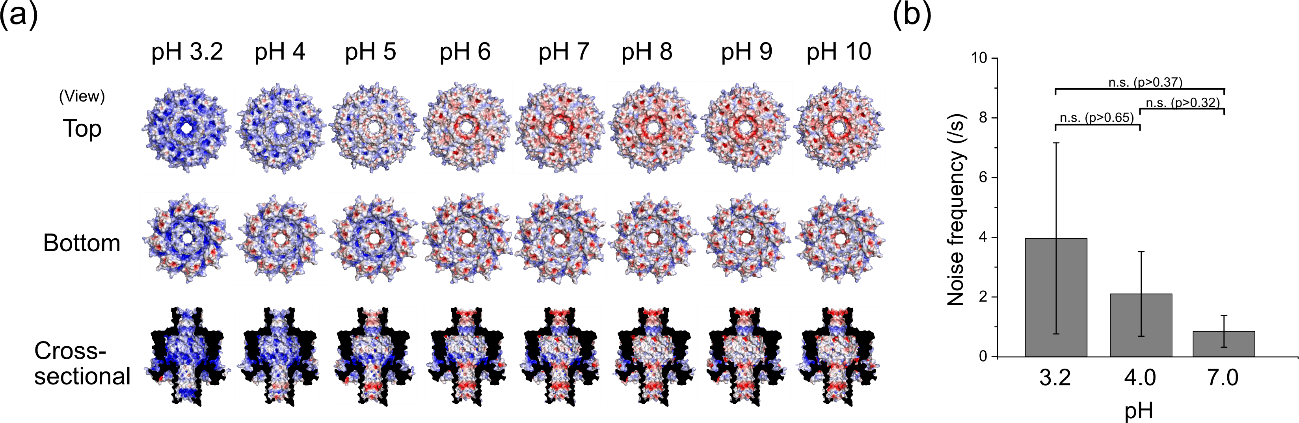


**Figure S5. Pore properties of Epx4 nanopore in different pH conditions.** (a) Surface charge of Epx4 in different pH conditions. (b) Noise analysis of Epx4 in 1 M KCl at +50 mV at pH 3.2 (*N*=7, total open time: 85 s), 4.0 (*N*=5, total open time: 28 s), and 7.0 (*N*=12, total open time: 49 s). The bar graphs represent the mean ± SEM. *N* indicates the number of independent nanopores. Statistical analysis was conducted using Welch’s t-test. The channel current measurements at pH 3.2 were conducted in 1 M KCl, 10 mM citric acid, whereas those at pH 4.0 were conducted in 1 M KCl, 10 mM acetic acid.


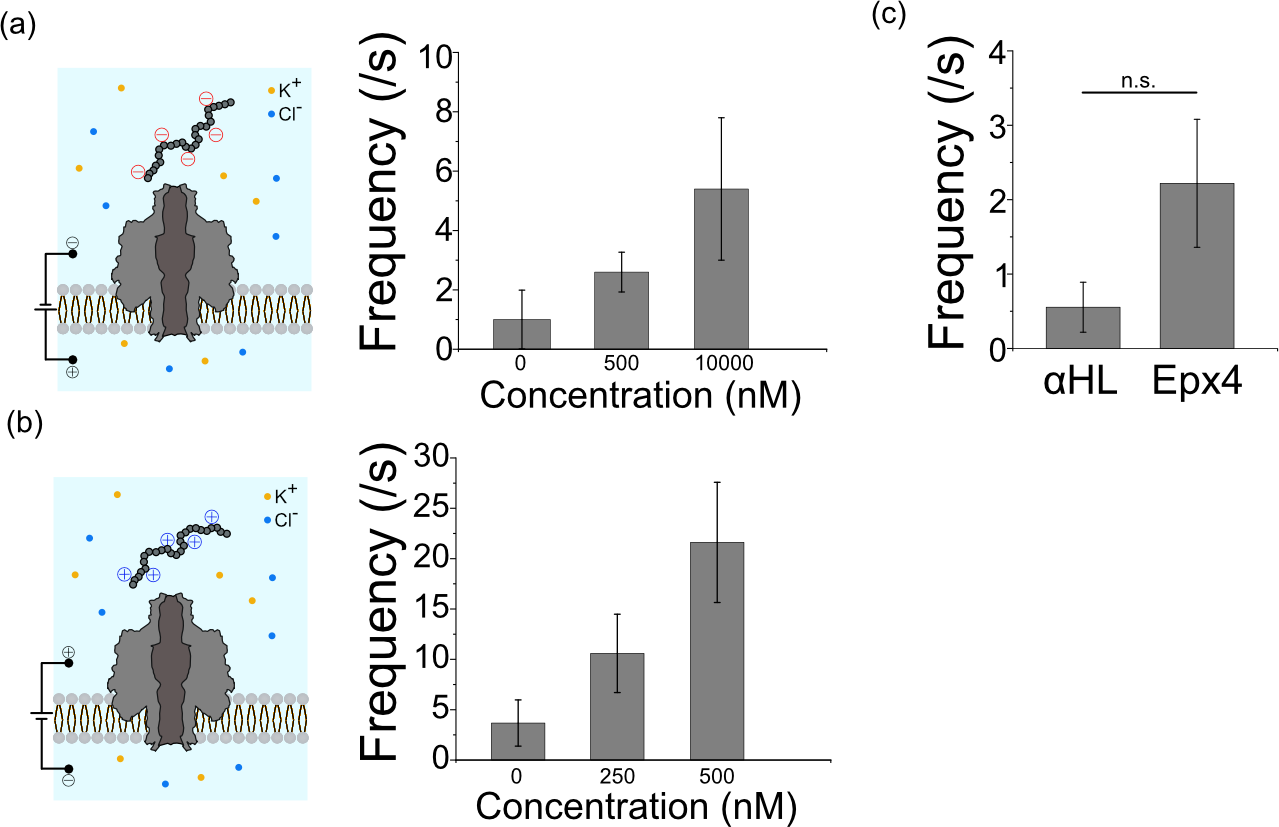


**Figure S6. Event frequency of polydT_50_ and S-PLL capture.** (a) Event frequency of polydT_50_ as a function of concentration. (b) Event frequency of S-PLL as a function of concentration. Epx4 produced blocking signals in the absence of the target molecules, which were regarded as false positives. Under the present detection conditions, these false positive signals could not be eliminated. (c) Comparison of the detection frequency of 500 nM ssDNA between Epx4 (*N*=4) and αHL (*N*=6). The bar graphs represent the mean ± SEM. *N* indicates the number of independent nanopores. Statistical analysis was conducted using Welch’s t-test (p>0.15). A two-sided Mann–Whitney U test was also performed and showed no significant difference (p>0.11).


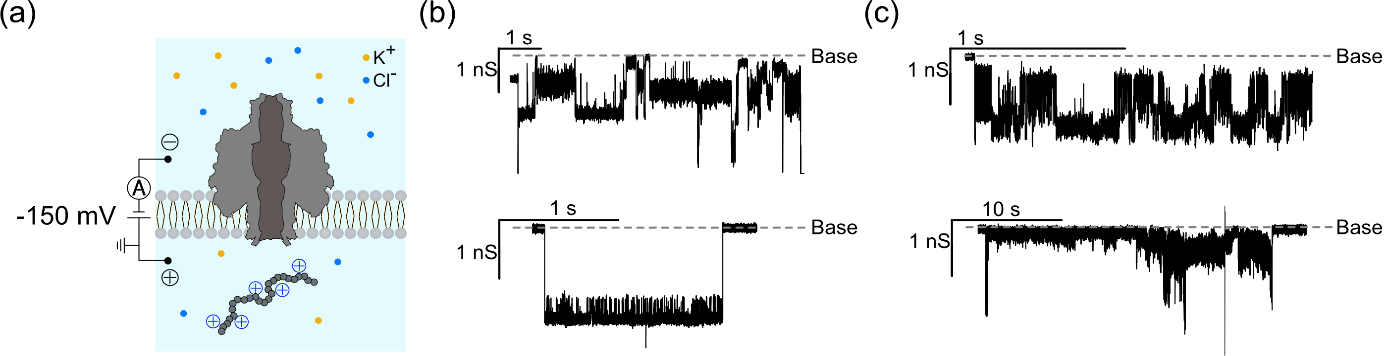


**Figure S7. S-PLL detection from the trans side of Epx4.** (a) Schematic diagram of detection experiments with S-PLL added from the trans side of Epx4 under the voltage of −150 mV. (b) Representative current traces showing signals longer than 1 s, which were observed in only 2 of 48 measurements. (c) Representative unstable current traces observed in the remaining measurements. The experimental difference between the cis and trans entry could not be directly investigated. As reported in αHL, electrostatic asymmetry may contribute to the directional difference in analyte capture (1).


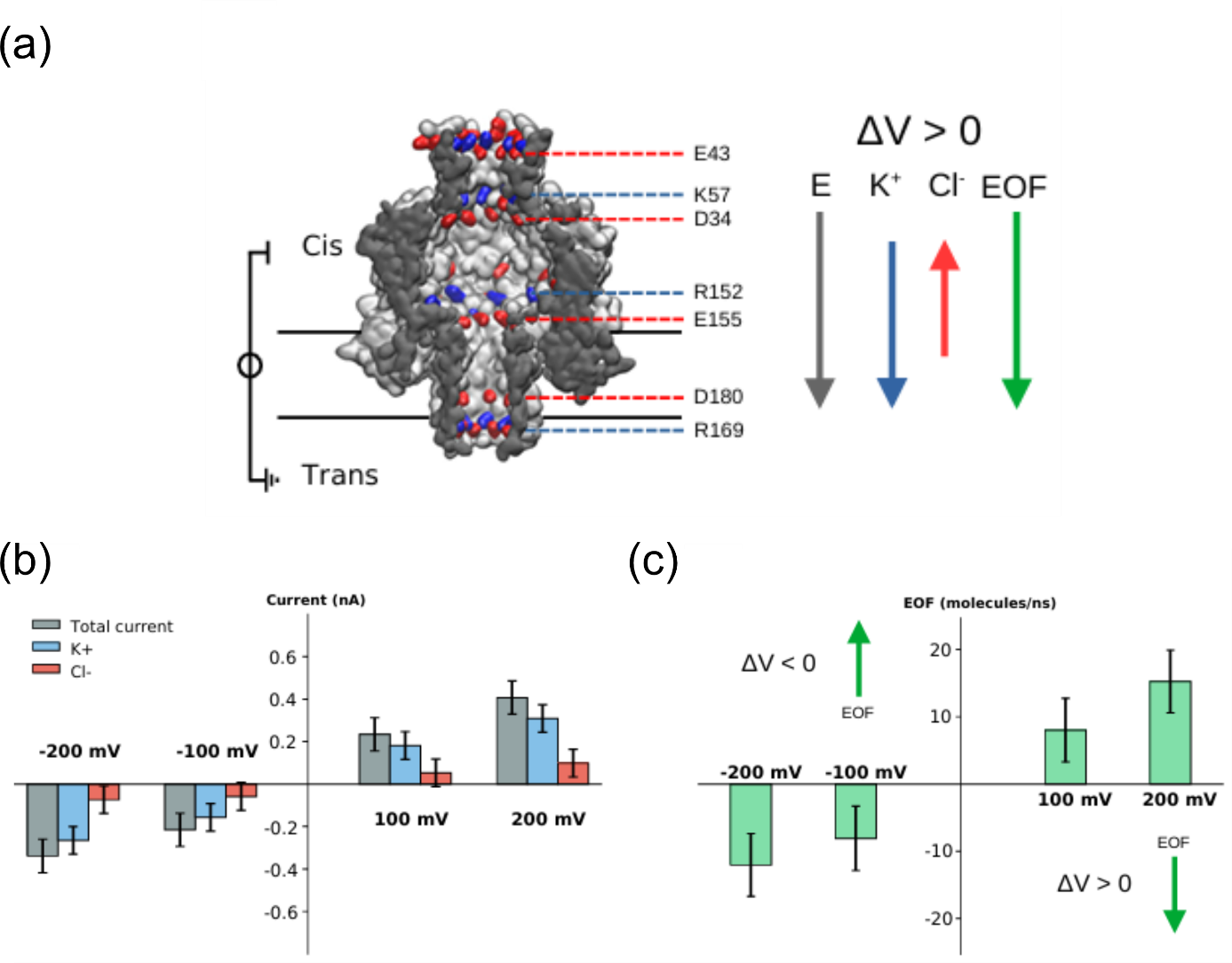
**Figure S8. Ion current and electroosmotic flow from MD simulation.** (a) Longitudinal section of wild-type Epx4. Positive and negative residues at pH 7 are represented in blue and red, respectively. Only residues exposed towards the pore lumen are shown. (b,c) Total, cationic and anionic currents (b) and electroosmotic flow (EOF) (c) for 1 M KCl as a function of applied voltage. The pore is cation-selective. In our convention the EOF is positive when water moves from cis to trans (indicated by the green arrows). Our simulations indicate that EOF is always directed as positive relative to the cation flux. For instance, at ∆*V* > 0, the electric field goes from cis to trans chamber, consequently, positive ions move in the same direction while negative ions move from trans to cis. The resulting EOF is from cis to trans favoring the capture of molecules located in the cis chamber. Panel (a) is drawn using VMD (3). Averages are calculated over a single time series and error bars represent standard errors estimated from statistically independent samples based on the correlation time.


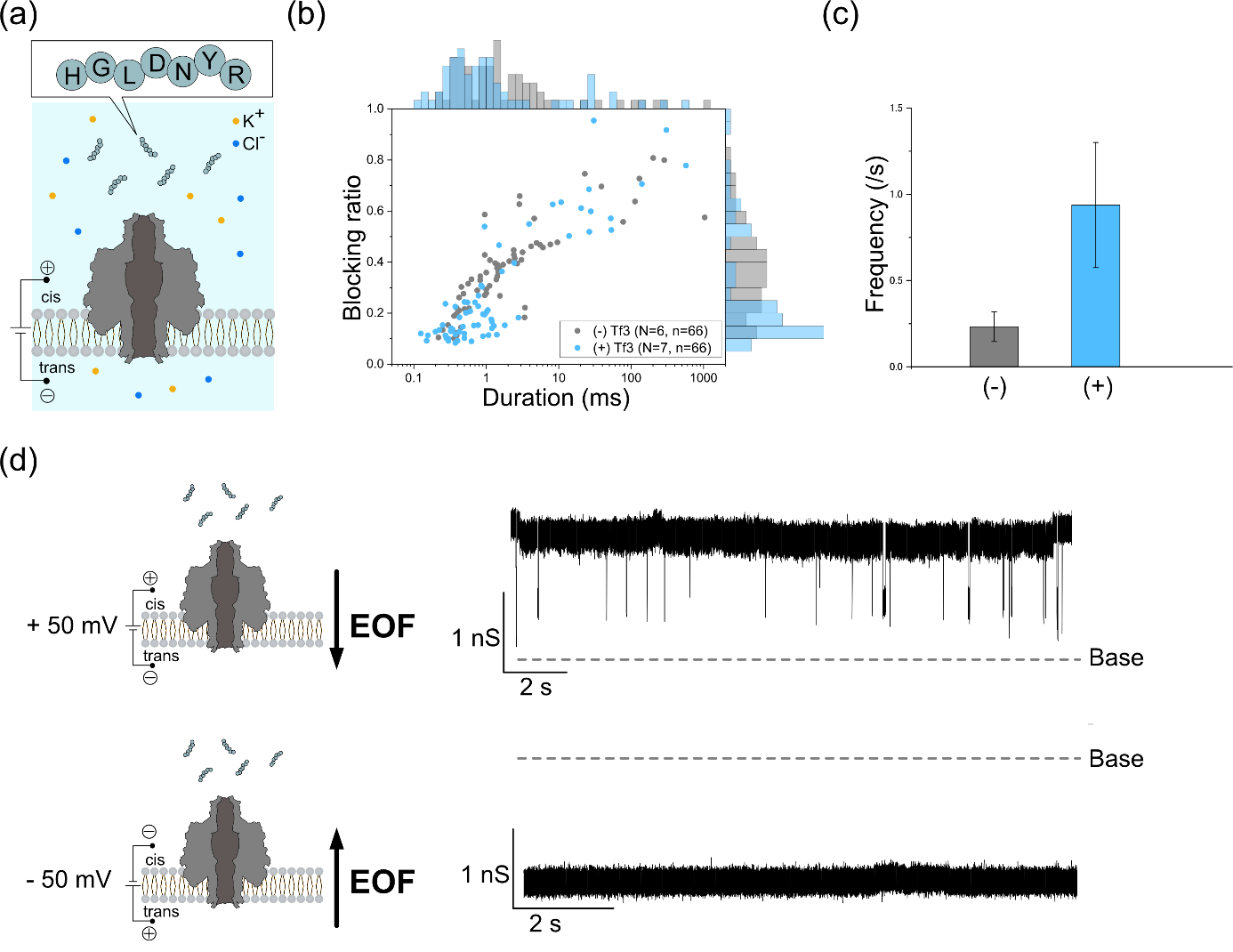


**Figure S9. Neutral peptide detection by using Epx4 nanopore.** (a) Schematic diagram of Tf3 detection through Epx4 nanopore. (b) Scatter plot of duration time vs blocking ratio. The plot obtained with Tf3 is colored blue and the plot obtained without Tf3 is colored gray. 66 blue data points were randomly selected to match the number of data points. (c) Comparison of the event frequency with and without Tf3. The bar graph shows mean ± SEM. These data were obtained from at least three independent nanopores. (d) Typical signals during voltage reversal. The blocking signals were observed at the EOF direction from the cis to the trans side, but few signals were observed when the EOF was directed from the trans to the cis side.


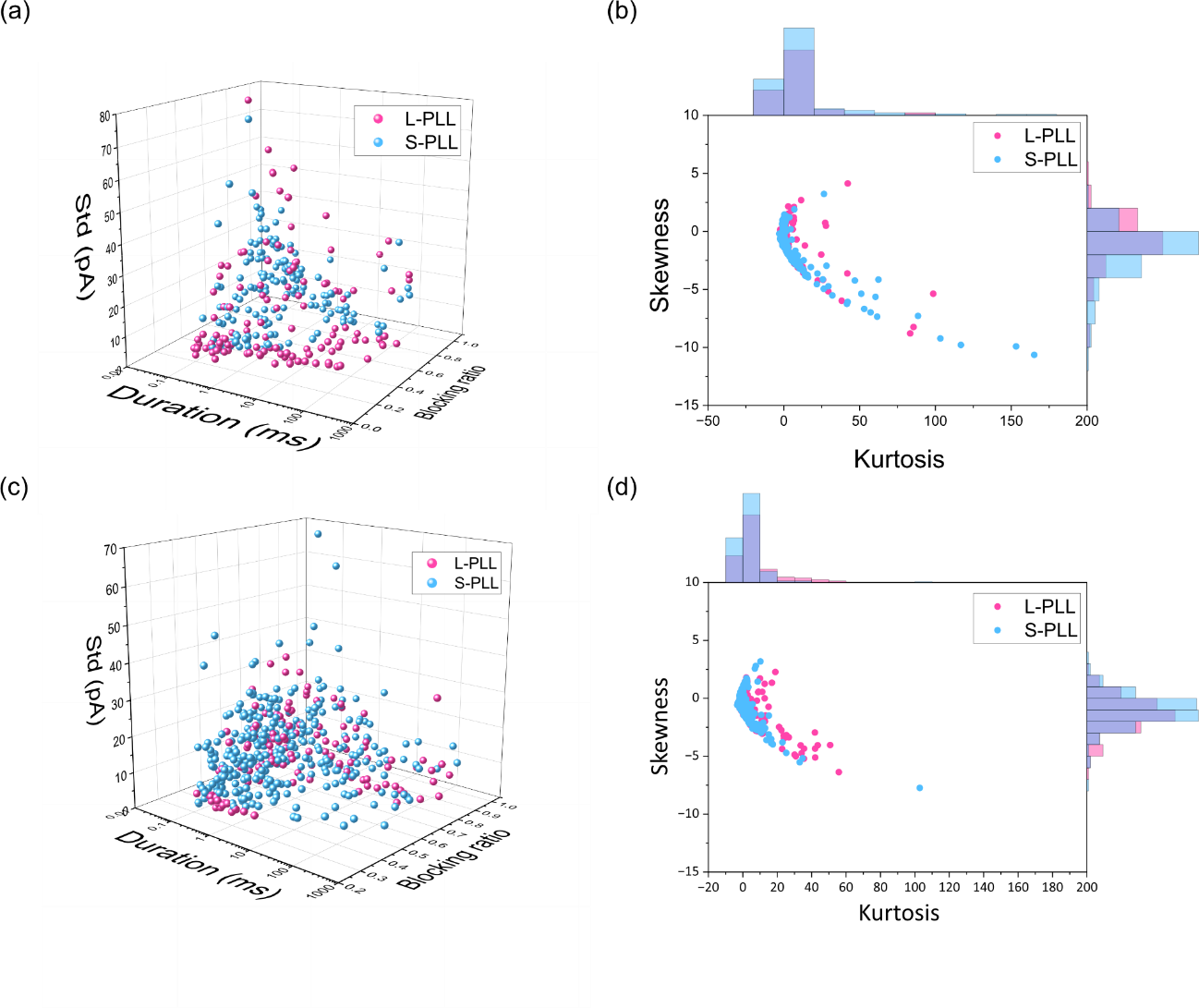


**Figure S10. Distribution of each feature for PLLs.** (a) Scatter plot of duration time vs. blocking vs. Std in Epx4. (b) Scatter plot of kurtosis vs. skewness in Epx4. (c) Scatter plot of duration time vs. blocking vs. Std in αHL. (d) Scatter plot of kurtosis vs. skewness in αHL.


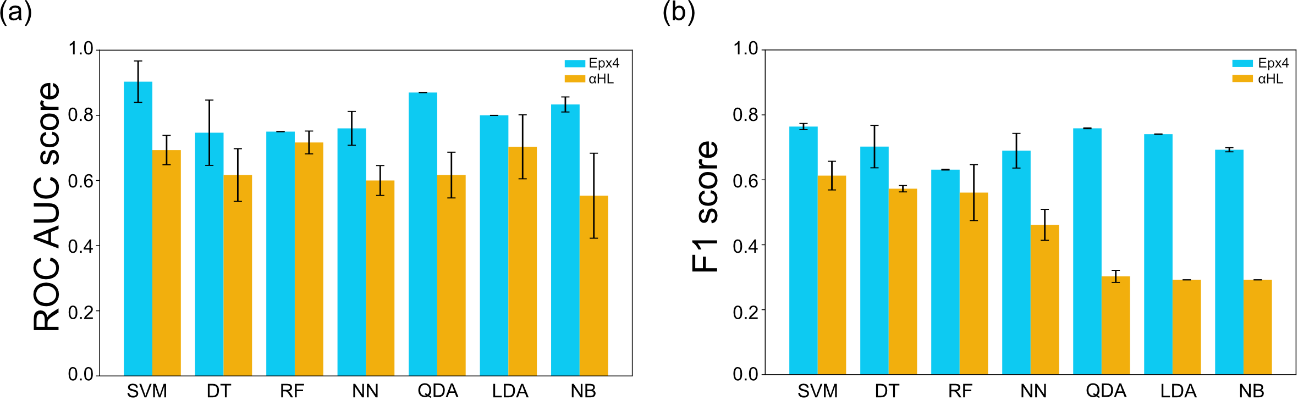


**Figure S11. Classifier performance across three times.** (a) ROC AUC and (b) F1 scores for each classifier. The bar graphs represent the mean ± SD across three different random seeds.

**References**

1. Xiong, X.; Tian, S.; Yang, P.; Lebreton, F.; Bao, H.; Sheng, K.; Yin, L.; Chen, P.; Zhang, J.; Qi, W. Emerging enterococcus pore-forming toxins with MHC/HLA-I as receptors. Cell 2022, 185 (7), 1157-1171. e1122.
2. Hille, B. Ion Channels of Excitable Membranes, 3rd ed.; Sinauer Associates, Inc.: Sunderland, MA, 2001.
3. Humphrey, W., Dalke, A., & Schulten, K. (1996). VMD: visual molecular dynamics. Journal of molecular graphics, 14(1), 33-38.
